# Late-onset severe biliary bleeding after endoscopic pigtail plastic stent insertion

**DOI:** 10.3748/wjg.v23.i4.735

**Published:** 2017-01-28

**Authors:** Muneji Yasuda, Hideki Sato, Yuki Koyama, Tomoki Sakakida, Takumi Kawakami, Takeshi Nishimura, Hideki Fujii, Yoshikazu Nakatsugawa, Shinya Yamada, Naoya Tomatsuri, Yusuke Okuyama, Hiroyuki Kimura, Takaaki Ito, Hiroyuki Morishita, Norimasa Yoshida

**Affiliations:** Muneji Yasuda, Hideki Sato, Yuki Koyama, Tomoki Sakakida, Takumi Kawakami, Takeshi Nishimura, Hideki Fujii, Yoshikazu Nakatsugawa, Shinya Yamada, Naoya Tomatsuri, Yusuke Okuyama, Hiroyuki Kimura, Norimasa Yoshida, Department of Gastroenterology and Hepatology, Japanese Red Cross Kyoto Daiichi Hospital, Kyoto 605-0981, Japan; Takaaki Ito, Hiroyuki Morishita, Department of Radiology, Japanese Red Cross Kyoto Daiichi Hospital, Kyoto 605-0981, Japan

**Keywords:** Biliary stent, Plastic stent, Biliary bleeding, Pseudoaneurysm, Pigtail stent

## Abstract

Here, we report our experience with a case of severe biliary bleeding due to a hepatic arterial pseudoaneurysm that had developed 1 year after endoscopic biliary plastic stent insertion. The patient, a 78-year-old woman, presented with hematemesis and obstructive jaundice. Ruptured hepatic arterial pseudoaneurysm was diagnosed, which was suspected to have been caused by long-term placement of an endoscopic retrograde biliary drainage (ERBD) stent. This episode of biliary bleeding was successfully treated by transarterial embolization (TAE). Pseudoaneurysm leading to hemobilia is a rare but potentially fatal complication in patients with long-term placement of ERBD. TAE is a minimally invasive procedure that offers effective treatment for biliary bleeding.

**Core tip:** Biliary bleeding after endoscopic pigtail plastic stent insertion is a rare but potentially fatal complication. Transarterial embolization (TAE) is a minimally invasive procedure that offers effective treatment for pseudoaneurysm. We report here a case of biliary bleeding caused by long-term placement of a pigtail plastic stent, which was inserted without removal of common bile duct stones due to the patient’s age; the TAE treatment was successful. This case report will help similar patients yet to be encountered but likely to increase in number due to ageing of the world’s population.

## INTRODUCTION

Biliary bleeding is an uncommon cause of upper gastrointestinal bleeding and biliary tract obstruction. Biliary stent-related biliary bleeding that occurs after an endoscopic procedure has been reported less often than biliary bleeding related to the percutaneous transhepatic procedure; however, its incidence has been increasing due to the general increase in endoscopic interventions. Transarterial embolization (TAE) is an effective treatment for traumatic pseudoaneurysm of visceral arteries. We describe, herein, a patient with gastrointestinal bleeding and biliary tract obstruction due to intrabiliary rupture of hepatic arterial pseudoaneurysm, which was successfully treated by TAE and percutaneous transhepatic biliary drainage (PTBD).

## CASE REPORT

### Patient

A 78-year-old woman.

### Chief complaint

Hematemesis.

### Past related medical history

**Figure 1:**
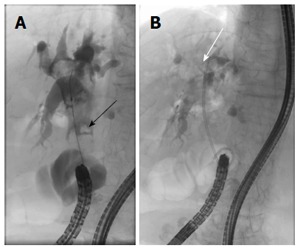
Endoscopic retrograde cholangiopancreatography image. A: Stones in common bile duct (black arrow); B: The plastic stent had been placed improperly, and the edge was coiled around in the common bile duct (white arrow). There was no sign of collapse or leakage in the biliary tract.

Thirty years before the current admission, the patient had undergone left hepatectomy, cholecystectomy and choledochectomy for intrahepatic and common bile duct (CBD) stones, in conjunction with Roux-en-Y and biliary reconstruction. At 1 year prior to the current admission, the patient had developed acute obstructive cholangitis due to CBD stones and had subsequently undergone single-balloon endoscopy. During that procedure, a 7 Fr pigtail type plastic biliary stent had been inserted into the CBD, which was already dilated due to the previous biliary reconstruction. The proximal edge of the plastic stent had been placed, coiled around, in the CBD improperly (Figure 1). After the cholangitis had been resolved, the patient refused removal of her CBD stones, citing age as her reasoning.

### Family history

We found nothing of significance in the patient’s family history.

### Case presentation

**Figure 2:**
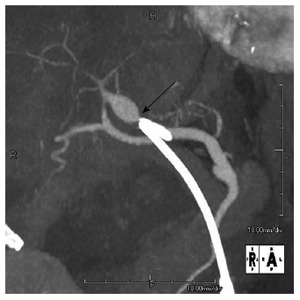
Abdominal computed tomography angiography. A 13 mm × 10 mm pseudoaneurysm was found at the proximal anterior segment of the right hepatic artery; The hepatic side edge of the plastic stent (black arrow) was close to the pseudoaneurysm.

**Figure 3:**
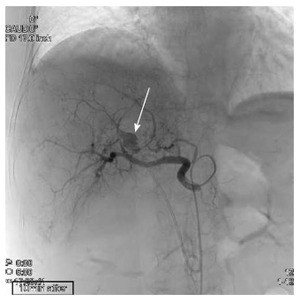
The pseudoaneurysm was detected upon celiac axis injection, with the aneurysmal sac slightly lateral to the edge of the plastic stent (arrow). The sac was no longer seen on a selective digital subtraction angiography image after successful embolization. Blood flow was maintained in the anterior segment of the right hepatic artery.

The patient presented to our emergency department with repeated episodes of hematemesis, which had occurred for 6 h. She was not on any medications. On examination, she had pallor of the palpebral conjunctiva, subfebrile temperature (37.3 °C) and tachycardia (heart rate of 99 bpm); her blood pressure was 14.4/5.9 kPa and her respiration rate was 20/min. She had no abdominal pain. Laboratory findings were as follows; hemoglobin of 9.6 g/dL (normal: 11.3-15.2 mg/dL); aspartate aminotransferase/alanine aminotransferase of 155/58 IU/L (normal: 13-33/2-27 IU/L); alkaline phosphatase of 1208 IU/L (normal: 115-359 IU/L); total bilirubin of 1.8 mg/dL (normal: 0.3-1.2 mg/dL); C-reactive protein of 2.6 mg/dL (normal: < 0.3 mg/dL); white blood cell count of 10180/μL (normal: 4000-8000/μL). Non-enhanced computed tomography (CT) revealed dilation of the CBD, as well as high-density masses in the bile duct and in the afferent intestinal loop. Single-balloon endoscopy of the small intestine revealed copious fresh coagulum in the afferent loop, but the site of bleeding was not detected. Contrast-enhanced CT showed 13 mm × 10 mm pseudoaneurysm of the hepatic artery, proximal to the edge of the plastic biliary stent (Figure 2). Abdominal angiography was performed and the pseudoaneurysm was detected close to the stent in the anterior segment artery of the right hepatic artery. N-butyl-2-cyanoacrlate was applied to embolize the pseudoaneurysm (Figure 3), after which a PTBD tube was inserted in the direction from B6 (the inferior branch of right posterior bile duct, corresponding to S6 of the Couinaud classification adopted by Bismuth) to the afferent loop. As a result, the patient’s jaundice improved and she was discharged on the 34^th^ hospital day.

**Figure 4:**
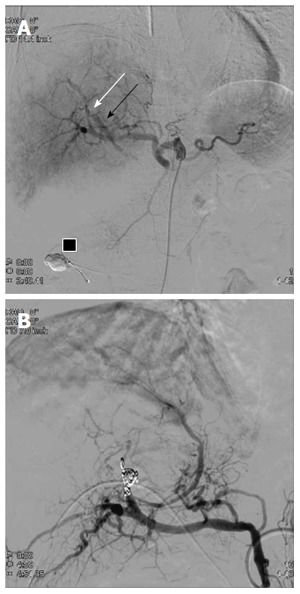
The 2^nd^ digital subtraction angiography image. A: Selective digital subtraction angiography image of the common hepatic artery showing an arterio-biliary fistula (white arrow) between A8 and the common bile duct (black arrow). Embolic material that migrated into the afferent loop is present (black box); B: Proximal A8 embolization obliterated the pseudoaneurysm. Leakage of contrast medium was completely controlled after embolization.

On the 3^rd^ day after discharge, the patient returned to the emergency department with hematemesis, jaundice and shock. Enhanced CT showed extravasation of contrast medium at the site of the pseudoaneurysm, suggesting that the embolic material may have migrated to the jejunum. Emergent abdominal angiography was performed and the digital subtraction angiogram (DSA) revealed an aneurysm at A8 (the anterior arterial branch supplying the superior subsegment) with an arterio-biliary fistula (Figure 4A). Collateral arteries to the anterior segment of the right lobe were also seen. Embolization was performed for A8, proximal to the pseudoaneurysm (Figure 4B). Subsequently, the patient developed an S8 (Couinaud classification) liver abscess, which was treated successfully by percutaneous transhepatic drainage.

The patient has survived for 1 year since final discharge, without any new health issues.

## DISCUSSION

Various reports about biliary bleeding have been published. Although biliary hemorrhage is relatively common after surgery, percutaneous transhepatic procedures and placement of the self-expanding metallic biliary stents[1-5], it is rare after insertion of a plastic stent. Likewise, only a few articles have reported on biliary bleeding associated with plastic stents[6-8].

We managed events of both life-threatening bleeding and acute obstructive suppurative cholangitis in a patient with a history of intestinal tract reconstruction. Due to this patient’s previous surgery and severe bleeding, endoscopic biliary drainage was relatively difficult, so we chose to perform PTBD for obstructive jaundice. We presume that this hepatic pseudoaneurysm might have formed as a result of traumatic stimulation related to the plastic stent placement because it had been placed improperly, with its tip located at the site of the aneurysm. Yet, this may not be the only cause. The patient’s history of cholangitis and CBD stones could have also contributed to the formation of the pseudoaneurysm. In daily clinical practice, an ERBD stent is usually placed for temporary drainage. However, we sometimes leave biliary stones and the ERBD stent in elderly patients due to their comorbidities, so it is not uncommon for us to see such patients[9].

In the study conducted by Green et al[10], cases of biliary bleeding, including those due to pseudoaneurysm, were mostly treated conservatively (43%) or by TAE (36%), with 20% undergoing surgery. In general, the preferred method to stop life-threatening biliary tract bleeding is TAE because it is less invasive and shows higher efficacy than the surgical approach[10]; moreover, TAE has lower reported rates of post-treatment mortality and morbidity than surgery[11,12]. However, cases of post-TAE hepatic artery occlusion and other complications, including fatal hepatic necrosis and intrahepatic abscess formation, have been reported[12-16]. Selective embolization of the vessel, as close as possible to the pseudoaneurysm, is desirable, both to reduce the risk of recurrent biliary bleeding and decrease the likelihood of hepatic necrosis[13].

For the patient described herein, we initially performed selective embolization and maintained peripheral flow because the pseudoaneurysm sac was detected by the first angiogram. Upon repeat angiography, an arterio-biliary fistula was found, instead of a sac, so we were forced to perform non-selective embolization of the anterior segment of the right hepatic artery. After the second TAE, the patient developed local liver necrosis and an intrahepatic abscess of S8, but she was successfully treated by percutaneous drainage and antibiotics. She has remained well for more than 1 year since final discharge.

In conclusion, hepatic artery pseudoaneurysm is a rare but potentially fatal complication in patients with a long-term placement of ERBD. TAE is a minimally invasive procedure that offers effective treatment for pseudoaneurysm.

## COMMENTS

### Case characteristics

A post-choledochectomy 78-year-old woman, with a pigtail biliary stent and CBD stones left in after previous treatment for cholangitis 1 year prior, presented with hematemesis.

### Clinical diagnosis

Biliary bleeding from a hepatic pseudoaneurysm and obstructive jaundice were detected by imaging examination.

### Differential diagnosis

Combination of peptic ulcer and biliary stent obstruction.

### Laboratory diagnosis

Elevated hepatobiliary system enzyme and C-reactive protein levels and leukocytosis suggestive of obstructive cholangitis. Anemia suggestive of bleeding.

### Imaging diagnosis

Contrast-enhanced computed tomography scan showed a 13 mm × 10 mm pseudoaneurysm of the hepatic artery, proximal to the edge of the plastic biliary stent.

### Treatment

Transarterial embolization (TAE) and percutaneous transhepatic biliary drainage.

### Related reports

Although biliary hemorrhage is relatively common after surgery, percutaneous transhepatic procedures and placement of self-expanding metallic biliary stents, it is rare after insertion of a plastic stent.

### Term explanation

The Couinaud classification is used to describe functional liver anatomy. It has emerged as the preferred anatomy classification system since it divides the liver into eight independent functional units.

### Experiences and lessons

The pigtail plastic stent is often used for temporary biliary drainage. However, it is important to remember that this stent type can cause fatal bleeding. TAE is a more effective and less invasive treatment for biliary bleeding than surgery.

### Peer-review

Although trauma caused by the proximal tip of the pigtail stent is highly suspicious, the authors explain that there might be other additional factors for the formation of the hepatic pseudoaneurysm.
